# Late toxicity and quality of life after definitive treatment of prostate cancer: redefining optimal rectal sparing constraints for intensity-modulated radiation therapy

**DOI:** 10.1002/cam4.261

**Published:** 2014-05-06

**Authors:** Sravana K Chennupati, Charles A Pelizzari, Rangesh Kunnavakkam, Stanley L Liauw

**Affiliations:** 1 Department of Radiation Medicine, Oregon Health & Science UniversityPortland, Oregon; 2 Department of Radiation and Cellular Oncology, Pritzker School of Medicine, University of ChicagoChicago, Illinois; 3 Department of Health Studies, University of Chicago HospitalsChicago, Illinois

**Keywords:** Dose–volume histogram, prostate cancer, quality of life, radiation therapy

## Abstract

The objective of this study was to assess late toxicity and quality of life (QOL) for patients receiving definitive intensity-modulated radiotherapy (IMRT) and image-guided radiation therapy (IGRT) with regard to normal tissue sparing objectives. Three hundred and seventy-two consecutive men treated with definitive IMRT for prostate adenocarcinoma. Toxicity was graded by CTC v3.0 genitourinary (GU) and gastrointestinal (GI) toxicity at each follow-up visit. Patient-reported QOL (EPIC-26) was prospectively collected for a subset of men. Dosimetric data for bladder and rectum were compared to toxicity and QOL global domain scores, specifically analyzing outcomes for men who met ideal rectal constraints (V70 <10%, V65 <20%, V40 <40%). The median age and prescription dose was 69 years and 76 Gy, respectively. Median follow-up was 47 months. At 4 years, freedom from Grade 2 (FFG2) GI toxicity was 92% and FFG2 GU toxicity was 76%. On univariate analysis, current smoking, larger bladder volume, and higher RT dose were associated with decreased FFG2 GU toxicity, while use of anticoagulation, increasing age, and not meeting ideal rectal constraints were associated with decreased FFG2 GI toxicity (all *P* ≤ 0.05). Bowel QOL remained stable over the 2-year follow-up period and was higher for patients who met ideal rectal constraints (*P* = 0.05). IMRT with IGRT is associated with low rates of severe toxicity and a high GI and GU QOL. The use of strict rectal constraints can further improve GI QOL and reduce GI toxicity.

## Introduction

Several randomized trials support the use of higher doses of radiation in the treatment of prostate cancer 1,2. However, the majority of these studies, which were conducted in the era of 2D and 3D treatment planning, also demonstrated increased risk of acute or late morbidity 2–4. Since the inception of these trials, prostate cancer radiotherapy has evolved. Intensity-modulated radiation therapy (IMRT), with daily image guidance (using ultrasound, prostatic fiducial markers, or cone beam CT), is commonly used in conjunction with normal tissue sparing goals. For example, rectal sparing objectives used in RTOG 0126 limited the volume of rectum receiving 70 Gy to <25%, and the volume of rectum receiving 65 Gy to <35%. A preliminary analysis of this study reported median values of 18% and 23%, respectively, with IMRT, and patients with a rectal V70 >15% experienced increased risk of late rectal toxicity 5. Notably, current technology can be used to achieve sparing goals which are more stringent, especially when pelvic lymph nodes are not included in the radiation field.

The primary goal of this study was to assess the effect of normal tissue constraints on late toxicity and quality of life (QOL) in the era of IMRT and image-guided radiation therapy (RT). A previous publication 6 sought to define optimal sparing goals and established a favorable outcome for men who met a rectal constraint that included V70 Gy <10%. With a larger cohort, longer follow-up, and data regarding patient-reported QOL, we hypothesized that patients who met this strict rectal constraint would have both lower rates of late gastrointestinal (GI) toxicity, and improved patient-reported QOL. Such data that are more consistent with current standards of therapy would be valuable in the counsel of prospective patients, and the planning practice of radiation oncologists.

## Methods

Between 2001 and 2010, 372 consecutive men were treated with curative intent IMRT for nonmetastatic adenocarcinoma of the prostate. Patients treated with prostatectomy or seed implant were excluded. Patients had a minimum of 24 months of potential follow-up, and were prospectively followed with all data coded into an institutional database. The collection and analysis of data were conducted with approval from the Institutional Review Board.

Patient and treatment characteristics are shown in Table1. The median age was 69 years. The median pretreatment prostate-specific antigen (PSA) was 8.3 ng/mL. In all, 147 (40%) men had a Gleason score ≤6, 168 (45%) men had Gleason 7, and 57 (15%) men had Gleason ≥8 disease. Only 34 men had cT3 disease (9%). By National Comprehensive Cancer Network (NCCN) risk category 7, 89 (24%) men were low risk, 175 (47%) men were intermediate risk, and 108 (29%) men were high risk. Androgen deprivation therapy (ADT) was given at the radiation oncologist's discretion based on the perceived risk benefit ratio of concurrent therapy, for a median of 4 months, to 198 (53%) men. Duration of therapy was ≤6 months in 128 men (of 174 with known duration, 74%). Prior to RT, 27 (7%) men underwent transurethral resection (TURP) of the prostate (median of 69 months prior to treatment). Thirty-nine (10%) men had taken anticoagulation therapy (warfarin or clopidogrel) either at consultation or during any follow-up visit.

**Table 1:** Patient and treatment characteristics (*n* = 372).

Characteristic	Number of patients (%, or range)
Median age (years)	69 (range, 47–88)
Median PSA (ng/mL)	8.3 (range, 1.5–658 ng/mL)
Gleason score	
≤6	147 (40)
7	168 (45)
8–10	57 (15)
Clinical stage	
cT1c	238 (64)
cT2	97 (26)
cT3	34 (9)
NCCN risk group	
Low	89 (24)
Intermediate	175 (45)
High	108 (29)
ADT	
Yes	198 (53)
No	174 (47)
Prior TURP	27 (7)
Anticoagulation	39 (10)
Radiation field design	
Prostate ± seminal vesicles	301 (81)
Pelvic lymph nodes with prostate boost	71 (19)

NCCN, National Comprehensive Cancer Network; ADT, androgen deprivation therapy; TURP, transurethral resection of prostate; SV, seminal vesicles; PSA, prostate-specific antigen.

### Radiation planning

Patient simulation and planning have been described in detail previously 8. Patients took a rectal enema 1–2 h prior to simulation. Patients were simulated supine, and alpha-cradle was used for immobilization. The bladder was drained and infused with 120 mL of contrast containing solution. For low-risk prostate cancer, the clinical target volume (CTV) was defined as the prostate alone. For intermediate- and high-risk prostate cancer, the initial clinical target volume was typically defined as the prostate plus proximal 2 cm of seminal vesicles (SV). In 71 (19%) men, the initial CTV also included pelvic nodes at risk 9. The use of pelvic nodal RT was reserved for men at the highest risk of lymph node involvement (e.g., those with clinical T3 disease). The planning target volume (PTV) expansion was 5–6 mm posteriorly and 8–10 mm otherwise. The prescription dose for PTV1 was 45–50.4 Gy at 1.8–2 Gy per fraction. PTV2 was treated an additional 24–30.6 Gy at 1.8–2 Gy per fraction. The median dose to PTV2 was 76 Gy (range, 69–79.2 Gy). Daily image guidance was used for all men after 2003, with transabdominal ultrasound (*n* = 177, 48%) or intraprostatic gold fiducial markers identified by kV imaging (*n* = 99, 27%). Treatment was delivered 5 days per week, via 6-MV photons with 7–9 coplanar fields with step-and-shoot IMRT. Normal tissue sparing goals were applied over the entire time period of study. The rectum was defined as a cylindrical structure around the outer rectal wall and contoured from the ischial tuberosities to the rectosigmoid junction. The bladder was defined by the outer bladder wall. IMRT planning constraints were tightened in 2007 as: PTV: V100% >95%, V95% >98%, V105% <10%, V110% <5%; rectum: V70 Gy <10%, V65 <20%, V40 <40%, bladder: V70 Gy <15%, V65 Gy <30%, V40 Gy <60%. Men who received treatment to an initial pelvic nodal field had relaxed normal tissue constraints including: rectum: V70 Gy <20%, V65 <40%, V40 <80%, bladder: V70 Gy <30%, V65 Gy <60%, V40 Gy <80%.

To further analyze differences between these groups, dosimetric data regarding the PTV, bladder, and rectum were analyzed for each patient from the original treatment plan and recomputed as cumulative dose–volume histogram (DVH) data. DVH data were available in digital format for all men and are summarized in Table2.

**Table 2:** Dosimetric data for all patients (*n* = 372).

	All patients (*n* = 372)	WPRT (*n* = 71)	No WPRT (*n* = 301)
Prescription dose (Gy)	76 (74.4–76)	76 (75.6–79.2)	76 (74–76)
V75 rectum (%)	7.0 (8.8–15.6)	8.8 (5.6–11.2)	6.7 (4.3–8.7)
V70 rectum (%)	11.7 (8.8–15.6)	13.7 (9.6–16.1)	11.2 (8.5–15.4)
V65 rectum (%)	16.2 (12.3–21.6)	18.7 (13.4–22.5)	15.9 (12.2–21.5)
V40 rectum (%)	54.1 (42.2–65.6)	65.0 (56.4–77.0)	51.4 (39.3–62.5)
Rectal mean dose (Gy)	43.9 (38.9–48.5)	47.7 (44.8–50.9)	42.9 (37.1–47.5)
Rectal volume (mL)	96.2 (80.1–121.2)	94.4 (77.3–118.7)	97.2 (80.2–122.0)
V75 bladder (%)	9.4 (5.9–12.7)	10.3 (6.9–13.3)	9.2 (5.6–12.6)
V70 bladder (%)	13.5 (9.2–18.4)	15.0 (10.9–19.2)	13.1 (8.9–18.4)
V65 bladder (%)	17.4 (12.0–24.0)	19.8 (14.1–25.4)	17.0 (11.8–23.4)
V40 bladder (%)	48.6 (35.5–63.4)	72.4 (61.3–80.1)	43.3 (32.5–54.2)
Bladder mean dose (Gy)	41.3 (33.1–48.4)	50.1 (47.5–53.0)	38.3 (31.2–44.3)
Bladder volume (mL)	171.1 (139.1–220.5)	181.6 (154.7–249.7)	168.1 (133.3–213.9)
Prostate volume (mL)	40.2 (29.8–56.9)	33.7 (26.9–48.6)	41.1 (30.4–58.1)
Prostate mean dose (Gy)	79.8 (78.0–80.6)	81.2 (78.8–81.7)	79.7 (78.0–80.3)

Data are reported as median (interquartile range). V75 rectum (%), volume of rectum receiving 75 Gy in terms of percentage of the entire rectal volume; WPRT, whole pelvis radiation therapy used for initial phase of treatment.

### Late toxicity

The median follow-up was 47 months from the end of RT to the last follow-up. Patients were seen 6–8 weeks after completion of RT and then every 6 months for the first 2 years. After 2 years, patients were seen in 6- to 9-month intervals until 5 years after therapy, at which point they were seen yearly. RTOG toxicity grades were assigned prospectively for genitourinary (GU) and GI toxicity at the date of each follow-up visit by the attending physician. More detailed Common Toxicity Criteria (CTC) version 3.0 toxicity grades were then assigned retrospectively by chart review. All toxicity reporting and analyses in this study involve CTC toxicity grade. Both GU (obstruction, incontinence, frequency, cystitis) and GI (diarrhea, proctitis, hemorrhage) systems were evaluated at each follow-up visit greater than 3 months after completing RT.

### Quality of life

Starting in 2007, patient-reported QOL data were prospectively collected and required for all clinic visits using the EPIC-26 tool 10. QOL data were obtained for men at time 0 (*n* = 86), 2 months (*n* = 100), 6 months (*n* = 107), 12 months (*n* = 120), 18 months (*n* = 128), and 24 months (*n* = 162 patients). The numbers increase over the follow-up period because men who were treated prior to 2007 still contributed QOL data. Scores were subdivided into three domains of urinary incontinence, urinary irritation or obstruction, and bowel or rectal dysfunction. Patients reporting distress or dysfunction (scored as a “moderate” or “big” problem) in each of the domains at the indicated time of follow-up were noted.

### Statistical analysis

Estimates of the freedom from Grade 2+ (FFG2) GI toxicity and FFG2 GU toxicity were calculated by the Kaplan–Meier method. Several clinical, disease, and treatment factors were tested against late toxicity in univariate (UVA) and multivariable analyses (MVA). UVA was performed for continuous variables after stratifying by the median value by log-rank test. The MVA model by Cox proportional hazards only included covariates with *P* < 0.1 on UVA. DVH parameters that were tested included the volume of normal tissue receiving 70 Gy, expressed as an absolute percent (V70), volume of normal tissue receiving 65 Gy (V65), volume of normal tissue receiving 40 Gy (V40), and overall volume of the rectum or bladder. In addition, our currently used set of rectal and bladder DVH parameters were also tested.

EPIC domain scores were normalized to 0–100 scale, with higher values representing a more favorable health-related QOL outcome. UVA was performed testing rectal and bladder constraints against the global bowel or urinary QOL score, respectively, using the log-rank test. QOL outcome measures were longitudinal in nature and were analyzed using the generalized estimation equations (GEE) to perform univariate and multivariate regression analysis in order to identify factors associated with changes in QOL over time. Separate GEE models were conducted for each QOL domain. All analyses were performed using GEE with patient as the grouping variable and with an exchangeable correlation structure. Only men with QOL data from a minimum of two time points were included in these analyses. This technique takes into account the within person correlation structure and provides robust standard errors 11.

## Results

### Characterization of late toxicity

For all patients, FFG2 GU toxicity was 86% at 2 years and 76% at 4 years. FFG2 GI toxicity was 94% at 2 years and 92% at 4 years. Late toxicity is detailed in Table3, including a breakdown of the type of GU or GI toxicity according to grade, and a comparison of toxicity at last follow-up and maximum toxicity at any point after therapy.

**Table 3:** Late toxicity (Common Toxicity Criteria 3.0) after IMRT to the prostate (*n* = 372), crude percentages over entire follow-up.

	Maximum toxicity (%)		At last follow-up (%)	
	≥Grade 2	≥Grade 3	≥Grade 2	≥Grade 3
Genitourinary				
Obstruction	4	2	2	1
Frequency	17	1	8	0
Cystitis	7	2	2	0
Incontinence	6	0	4	0
Gastrointestinal				
Hemorrhage	6	2	2	1
Proctitis	5	0	2	0
Diarrhea	2	0	1	0

IMRT, intensity-modulated radiotherapy.

### Univariate and multivariable analysis of late toxicity

On UVA, anticoagulation (*P* = 0.05), older patient age (*P* = 0.05), and failure to meet the triad of ideal rectal constraints (*P* = 0.03) were associated with inferior FFG2 GI toxicity. ADT use (*P* = 0.20), smoking history (*P* = 0.56), rectal volume (*P* = 0.89), prostate volume (*P* = 0.81), RT dose (*P* = 0.32), WPRT (*P* = 0.28), DM (*P* = 0.91), rectum V70 Gy (*P* = 0.09), rectum V65 Gy (*P* = 0.10), and rectum V40 Gy (*P* = 0.11) were not associated with FFG2 GI toxicity. Analysis of FFG2 GI toxicity at 4 years using the single DVH metric of V70 Gy rectum showed rates of 94% if V70 <10%, 94% if 10 < V70 ≤ 15%, 87% if 15 < V70 ≤ 20%, and 90% if V70 >20% (*P* = 0.30). Fifty-eight patients met optimal rectal planning criteria (V70 Gy <10%, V65 <20%, V40 <40%). For this group, FFG2 GI toxicity was 100% at 4 years compared to 93% for those that did not meet these criteria (*P* = 0.03). Meeting the optimal rectal constraints was associated with less proctitis (*P* = 0.05) and hemorrhage (*P* = 0.04). On multivariable analysis (MVA), use of anticoagulation (HR 2.99; 95% CI, 1.10–6.91; *P* = 0.03) and ideal rectal constraints (HR and 95% CI not calculated due to low number of events) were associated with FFG2 GI toxicity, while age was not (HR 2.00; 95% CI, 0.95–4.48; *P* = 0.07).

On UVA, smoking during treatment (*P* = 0.01), increased bladder volume (*P* = 0.01), and higher RT dose (*P* = 0.01) were associated with inferior FFG2 GU toxicity. ADT (*P* = 0.42), smoking history (*P* = 0.17), WPRT (*P* = 0.10), prostate volume (*P* = 0.64), anticoagulation (*P* = 0.70), age (*P* = 0.64), prior TURP (*P* = 0.77), DM (*P* = 0.24), strict bladder constraints (*P* = 0.60), bladder V70 Gy (*P* = 0.21), bladder V65 Gy (*P* = 0.17), and bladder V40 Gy (*P* = 0.11) were not associated with FFG2 GU toxicity. On MVA, smoking during treatment (RR 1.75; 95% CI, 1.08–2.75; *P* = 0.02) and bladder volume (RR 1.66; 95% CI, 1.10–2.54; *P* = 0.02) were associated with FFG2 GU toxicity. Results of the UVA and MVA are shown in Table4.

**Table 4:** Univariate and multivariate analysis and freedom from Grade 2 or higher gastrointestinal and genitourinary toxicity (*n* = 372).

	Gastrointestinal toxicity			Genitourinary toxicity		
	2 y FF G2+ toxicity	UVA *P*-value	MVA *P-*value	2 y FF G2+ toxicity	UVA *P-*value	MVA *P-*value
ADT, yes vs. no	96 vs. 92	0.20		84 vs. 89	0.42	
Smoking history, never vs. prior	96 vs. 94	0.56		84 vs. 86	0.17	
Smoking history, current vs. not current	94 vs. 96	0.29		87 vs. 82	0.01	0.02
Rectal volume, ≤ vs. > 96 cc	95 vs. 93	0.89				
Bladder volume, ≤ vs. > 171 cc				88 vs. 84	0.01	0.02
Prostate volume, ≤ vs. > 40 cc	94 vs. 94	0.81		87 vs. 91	0.64	
RT dose, ≤ vs. > 76 Gy	93 vs. 97	0.32		89 vs. 77	0.01	0.07
WPRT, no vs. yes	94 vs. 97	0.28		87 vs. 80	0.10	0.92
Anticoagulation, no vs. yes	94 vs. 92	0.05	0.04	86 vs. 84	0.70	
Age, ≤ vs. > 69 years	96 vs. 92	0.05	0.06	84 vs. 88	0.64	
TURP, yes vs. no				83 vs. 86	0.77	
Diabetes, no vs. yes	94 vs. 93	0.91		87 vs. 82	0.24	
Achieved strict OAR constraints^1^	100 vs. 93	0.03	0.01	86 vs. 86	0.60	
OAR V70 Gy, ≤ vs. > median	95 vs. 93	0.09	0.44	84 vs. 88	0.21	
OAR V65 Gy, ≤ vs. > median	95 vs. 93	0.10	0.70	83 vs. 89	0.17	
OAR V40 Gy, ≤ vs. > median	95 vs. 93	0.11		83 vs. 89	0.11	

ADT, androgen deprivation therapy; WPRT, whole pelvis radiation therapy; TURP, transurethral resection of prostate; OAR, organ at risk (i.e., rectum, bladder).

1 Rectum: V70 Gy <10%, V65 <20%, V40 <40%, bladder: V70 Gy <15%, V65 Gy <30%, V40 Gy <60%.

### Patient-reported QOL

**Figure 1:**
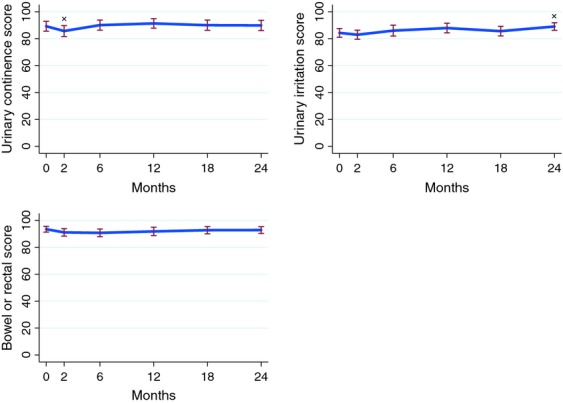
Global domain scores following radiation therapy (*n* = 86). The asterisk represents scores that were statistically different (*P* < 0.05, *t* test) to the baseline value (designated at 0 months). Standard error is shown by the vertical red lines.

QOL data were available for a subset of men and subdivided into domains of urinary irritation or obstruction, urinary incontinence, and bowel function. Urinary irritation or obstruction scores remained similar or were improved over the 24-month follow-up period. Urinary continence declined 2 months posttreatment, but this resolved by 6 months and was similar to baseline at 24 months. Bowel-related QOL remained stable for the 24-month period. A graphical representation of these results is shown in Figure1.

Tables5 and 6 list the percentage of all patients, or of patients meeting strict rectal planning constraints, with distress or dysfunction at different times of follow-up, respectively. For all patients, there was little change in bowel function from the baseline percentage (2–4%) over the 24-month period. For patients meeting strict rectum planning constraints, 2% of patients reported bowel distress or dysfunction 24 months after treatment. Overall bowel function QOL scores at 24 months were higher for the group of patients that met the triad of ideal rectal constraints compared to those who did not (median value 100 vs. 96; *P* = 0.05). A multivariable model including ideal rectal constraints, age, and anticoagulation against patient-reported, global bowel function use indicated that meeting rectal constraints was associated with bowel function (coefficient 4.7, CI 1.0–8.5, *P* = 0.01), while age (coefficient 0.17, CI −0.04 to 0.4, *P* = 0.11) and anticoagulation use (coefficient −1.6, CI −6.8 to 3.6, *P* = 0.55) were not.

**Table 5:** Percentage of all patients treated with IMRT reporting distress or dysfunction in each quality of life domain.

Variable	Baseline (*n* = 86)	2 months (*n* = 100)	6 months (*n* = 107)	12 months (*n* = 120)	18 months (*n* = 128)	24 months (*n* = 162)
**Urinary function**						
Irritation or obstruction						
Dysuria	1	4	6	2	2	1
Hematuria	2	2	1	1	2	1
Weak stream	9	5	8	10	5	8
Nocturia	23	18	21	22	18	15
Frequency	28	18	11	14	10	9
Incontinence						
Leaking >1 time per day	13	11	8	6	7	11
Frequent dribbling	6	4	5	4	6	6
Any pad use	2	8	6	4	6	4
Leaking problem	5	2	2	3	2	5
Overall urinary problem	19	12	11	12	11	9
**Bowel function**						
Urgency	2	6	5	4	3	3
Frequency	2	5	3	2	1	2
Fecal incontinence	0	1	2	3	1	1
Bloody stools	0	1	1	2	2	2
Rectal pain	1	3	2	0	2	3
Overall bowel problem	2	4	2	4	2	4

**Table 6:** Percentage of patients meeting rectal V70 Gy <10%, V65 <20%, V40 <40%, reporting distress or dysfunction for the bowel function quality-of-life domain.

Variable	Baseline (*n* = 32)	2 months (*n* = 36)	6 months (*n* = 39)	12 months (*n* = 39)	18 months (*n* = 37)	24 months (*n* = 45)
Bowel function						
Urgency	0	0	0	3	3	0
Frequency	0	6	3	0	0	2
Fecal Incontinence	0	0	3	3	0	0
Bloody stools	0	3	3	0	0	0
Rectal pain	3	6	3	0	0	2
Overall bowel problem	6	3	3	0	3	2

## Discussion

In this series, we report late toxicity after treatment for prostate cancer with IMRT, and investigate relationships between normal tissue radiation dose and physician-reported toxicity as well as patient-reported QOL. Our data demonstrate that dose-escalated RT can be associated with a low risk of severe late GI and GU toxicity, without adverse impact on urinary or bowel QOL at 2 years. When dose escalated IMRT is planned with strict rectal constraints (V70 Gy <10%, V65 <20%, V40 <40%), the rates of Grade 2+ GI toxicity are especially low (FFG2 GI toxicity 100% at 4 years) and the patient-reported GI QOL is very high. These results suggest that there is value in meeting a tighter rectal sparing goal (achievable with IMRT), than the more commonly accepted rectal sparing constraint of V70 Gy <15–25%. In addition, meeting tighter constraints my minimize the “negative” factors of advanced age or anticoagulation. Of note, use of V70 Gy <10% as a single metric alone was not associated with Grade 2+ GI toxicity, suggesting that the use of multiple points on the DVH curve may be important. While we did not identify any bladder constraints associated with reduced GU morbidity, urinary QOL was nevertheless stable after therapy, and urinary irritation or obstruction scores were even improved for the overall cohort at 2 years of follow-up. This improvement in obstructive symptoms is consistent with other reports 12 and could be a result of treatment itself 13, the use of medication, or patient adaptability toward urinary habits.

Given that the majority of men with prostate cancer who undergo local therapy will die of other causes 14, it is paramount to minimize morbidity and preserve QOL in men who receive treatment. A few patient-reported QOL studies after external beam RT have been published. One of the largest series detailing QOL after local therapy to the prostate was published in 2008 12. In this multi-institutional series, 292 patients received radiotherapy with IMRT (83%) or 3D conformal techniques (17%) to a prescribed dose of 75.6–79.2 Gy. The median rectal V70 Gy was 12% (interquartile range, 9–17). By the EPIC survey, all bowel function domains were adversely affected at 2 years, with 11% of patients reporting a moderate or severe “overall bowel problem.” A subsequent analysis of this multi-institutional cohort identified that rectal V70 ≥25% was associated with an inferior bowel QOL score and increased risk of fecal incontinence 15. QOL data from the Proton Radiation Oncology Group randomized trial of 70.2 or 79.2 Gy have also been reported, using Prostate Cancer Symptom Indices 16. Overall, there was no decline in GI or GU QOL after therapy. In a subset analysis of 50 men from this trial, higher dose to the anterior rectal wall (V60, V65, V70, and V75) was associated with inferior late GI QOL (minimum follow-up, 7 years), although higher prescription RT dose to the prostate itself was not 17. It is unclear whether the favorable late QOL in this series is at least partially attributed to the use of proton therapy. More recently, QOL after proton therapy, IMRT, or 3D conformal RT has been described. In this study, clinically meaningful differences were noted for all three modalities at 2 years for GI QOL, but not GU QOL 18. A prospective study of 227 men treated with IMRT (31%, 78 Gy) or 3D conformal therapy (69%, 70 Gy) was conducted using the EORTC QLQ-C30, and found that bowel QOL was adversely affected at 1 year, although this difference resolved by 2–3 years 19. Finally, follow-up of 194 men treated with 3D conformal RT to a mean dose of 74 Gy demonstrated a decline in bowel QOL by EPIC scores throughout 5 years of follow-up 20. To place our results into context, we demonstrate no decline in GI or GU QOL at 2 years of follow-up. At 2 years, moderate or severe bowel distress was reported in 4% of patients treated with IMRT in our cohort (2% in those meeting strict rectal planning constraints, which was no higher than baseline), and overall urinary distress was reported in 9% (compared to 19% at baseline). These early results are encouraging for the ability of image-guided IMRT to minimize QOL impairment that otherwise has been reported with other forms of external beam RT.

There are some limitations of this single-institution series. While this longitudinal study does extend over a long time frame with some heterogeneity in treatment over that time, we chose to include all patients to increase analytical power for DVH/toxicity analysis, and take advantage of the full dataset of physician-reported toxicity which was prospectively coded at each clinic visit. The slight heterogeneity in planning may actually increase the ability to identify important DVH relationships by providing a wider range of dose to the normal tissues. While the patient-reported QOL data are not as robust as the physician-reported toxicity in this series, analysis of QOL is still of value to further support the benefit in meeting strict rectal constraints. Additionally, the QOL data have practical value in guiding expectations of men post-treatment in era of image-guided IMRT. Longer follow-up beyond 2 years is necessary to demonstrate maintenance of a high QOL after therapy, especially for GU toxicity, which has been shown to increase with follow-up. Baseline QOL data were not available for all patients in our cohort, which reduces the statistical certainty of our analyses. However, the use of the GEE modeling does account for the change in patient numbers over time and an analysis that excluded patients that did not have baseline data available did not alter the conclusions. Finally, the introduction of stricter normal tissue parameters occurred toward the latter part of the study period, and nearly all men who did not receive pelvic nodal RT met these criteria after implementation. There is potential for bias in this more recently treated cohort, related to shorter follow-up and possibly refined planning or delivery techniques. However, by forcing a 2-year minimum follow-up time for all patients, we sought to minimize this bias on QOL. Meanwhile, since severe rectal toxicity typically develops within 1–2 years after RT, the chance for this bias to underreport GI toxicity should be small.

In summary, after image-guided IMRT to the prostate, rates of severe toxicity are low, and GI and GU QOL remain high. Our results support the use of strict rectal sparing guidelines when planning IMRT for prostate cancer. Patients who met the most strict set of rectal constraints (including V70 Gy <10%) had a lower risk of late GI morbidity and higher GI QOL. We recommend using rectum V70 Gy <10%, V65 <20%, V40 <40% when targeting the prostate and seminal vesicles, and V70 Gy <20%, V65 <40%, V40 <80% when including pelvic lymph nodes. Our patient-reported QOL results can be a useful guide to advice men who are considering different local therapies, as they reflect the ability for current technology to minimize adverse effects of therapy.

## Conflict of Interest

None declared.
